# An Anionic Porous Indium-Organic Framework with Nitrogen-Rich Linker for Efficient and Selective Removal of Trace Cationic Dyes

**DOI:** 10.3390/molecules28134980

**Published:** 2023-06-25

**Authors:** Lihui Feng, Xiaofei Zhang, Zhekuang Jin, Jiashang Chen, Xing Duan, Shiyu Ma, Tifeng Xia

**Affiliations:** 1 Center of Advanced Optoelectronic Materials and Devices, Key Laboratory of Novel Materials for Sensor of Zhejiang Province, College of Materials & Environmental Engineering, Hangzhou Dianzi University, Hangzhou 310018, China; 2 Institute of Materials, China Academy of Engineering Physics, Mianyang 621907, China

**Keywords:** indium-organic framework, anionic, selective removal, trace cationic dyes

## Abstract

Metal-organic frameworks (MOFs) with porosity and functional adjustability have great potential for the removal of organic dyes in the wastewater. Herein, an anionic porous metal-organic framework (MOFs) [Me_2_NH_2_]_2_In_2_[(TATAB)_4_(DMF)_4_]·(DMF)_4_(H_2_O)_4_ (HDU-1) was synthesized, which is constructed from a [In(OOC)_4_]^−^ cluster and a nitrogen-rich linker H_3_TATAB (4,4′,4″-*s*-triazine-1,3,5-triyltri-*p*-aminobenzoic acid). The negatively charged [In(OOC)_4_]^−^ cluster and uncoordinated –COOH on the linker result in one unit cell of HDU-1 having 8 negative sites. The zeta potential of -20.8 mV dispersed in pure water also shows that HDU-1 possesses negatively charged surface potential. The high electronegativity, water stability, and porosity of HDU-1 can facilitate the ion-exchange and Coulombic interaction. As expected, the HDU-1 exhibits high selectivity and removal rates towards trace cationic dyes with suitable size, such as methylene blue (MB) (96%), Brilliant green (BG) (99.3%), and Victoria blue B (VB) (93.6%).

## 1. Introduction

As raw materials for papermaking, printing and dyeing, textile, and other industries, organic dyes with certain toxicity are often discharged with industrial wastewater, which not only causes harm to the natural ecological environment, but also seriously threatens human health. In fact, the United Nations has chosen “Clean water and sanitation” as one of the Sustainable Development Goals for 2030. However, most organic dyes are difficult to remove from wastewater because of excellent chemical stability. Furthermore, some dyes can form toxic, mutagenic, and carcinogenic intermediates through hydrolysis and oxidation reactions [1,2]. Therefore, the adsorption and removal of dyes before discharge is particularly important for the purpose of the protection of human health and ecological environments. Many organic dye treatment technologies, such as adsorption, membrane separation, photocatalytic degradation, biocatalytic degradation, microwave irradiation, etc., have been widely used in wastewater treatment [3,4]. Among these technologies, adsorption is taken into account to be one of the most feasible and economical methods to deal with organic dye water pollution. However, traditional adsorbents, such as active carbon, zeolite, and silica, show weak adsorption efficiency and low selective separation toward trace dye molecules, which greatly limits their further practical application [5,6]. Therefore, the exploitation of novel and efficient adsorbents for the removal of trace dyes is urgent and necessary.

As a new class of porous organic-inorganic hybrid material, metal-organic frameworks (MOFs) are constructed from metal ions/clusters and organic linkers through coordination bond [1,2,3,4,5,6,7,8,9,10,11,12,13,14,15,16,17,18,19,20,21,22,23,24,25,26,27,28,29]. There are currently about 100,000 MOFs in the Cambridge database, far more than the types of silica and zeolite (1000). Compared to traditional porous materials, the diversity of metal ions/clusters and linkers results in MOFs displaying large specific surface areas, high porosity, structural designability, and surface functionability, already known as outstanding candidates for dye pollutions removal [1,4,6,7,8,9,10,11,12,13,14,15,16,17,18,19,20,21,22,23,24,25,26,27,28,29]. Choe et al. [1] reported that PCN-224 possesses the high BET surface area of 2982 m^2^/g and the large pore size distribution of 2.4 nm. Therefore, PCN-224 affords the highest adsorption capacity of 1015.7 mg/g for MO (methyl orange) molecules due to the π-π stacking interactions and hydrogen bonds. In addition to BET and pore size, the water stability also plays an important role in the use of wastewater application. Zhang et al. [7] utilized Cu(II) and a bifunctional tripodal nitrogen-donor linker to construct a MOF SCNU-74 with a cage cavity and channels. SCNU-74 exhibits high stability in water solution with a pH range from 3 to 11, and best adsorption capacity of 1200 mg/g toward CR (Congo red) because of the hydrogen bonds and the π-π stacking interaction between the framework of SCNU-74 and CR guest. At present, some related research has made breakthroughs and aroused wide interest of researchers.

Specifically, ionic-functional MOF materials display more invincible performance in the highly efficient elimination of pollutants and chemicals, especially dyes. This can be attributed to several factors: (1) Coulombic interaction and ion-exchange [11,12,13,14,15,16,17,18,19,20,21,22,23,24,25,26,27,28,29], which can promote the dyes with the opposite charge into the pore/channel of ionic MOFs, while the dyes with similar charge are repelled, then achieve selective separation. Zhao et al. [12] utilized the anionic zinc-organic framework which exhibited highly selective adsorption of methylene blue dye by the ion exchange mechanism. A functional anionic MOF [18] can efficiently remove the carcinogenic Basic Violet 14 (BV14) and Basic Red 9 (BR9) in conjunction with Coulombic interaction, hydrogen bonding, and ion-exchange. (2) Size-exclusion and high porosity. Dyes with different size and molecular weight can be selectively adsorbed by adjusting the pore size of MOFs. For example, Deng et al. [11] synthesized a cationic MOF with tubular channels based on Ni(II) ions and nitrogen-containing ligands, which exhibited excellent adsorption capacity for hazardous anionic contaminants with different sizes from few minutes to about 1 h in water environment. In addition, the results show that only cationic dye rhodamine B with suitable size can be removed due to a size-selectively adsorption. (3) Specific functional sites are introduced into the structure of MOFs to enhance the interaction of host-guest molecules and improve the adsorption capacity for dyes. Morsali et al. [24] applied “cavity functionalization” approach to achieve optimized host-guest interactions for TMU-34. TMU-34 with dihydro-tetrazine groups can efficiently remove rose-bengal B (RB-B) from aqueous solution by hydrogen bonding between free phenolate/carboxylate of RB-B and dihydro-tetrazine of TMU-34.

With a view to designing ionic MOFs, metal clusters are considered first. Nowadays, the In^3+^ sites have been widely utilized to construct ionic-functional MOFs because of its tendency to charge secondary building units (SBUs), such as positively charged [In_3_O(OOC)_6_]^+^ trigonal planar trimers [29,30,31], negatively charged [In(OOC)_4_]^−^ monomers [32,33], and [In_2_(OH)] dimers [34]. Aside from the metal clusters with an electric charge in MOFs, some organic ligands can also further provide a negative charge due to uncoordinated carboxyl groups or exposed functional groups, such as -COOH, -OH, imide groups. These functional groups can also enhance the interaction with dye molecules through hydrogen bonding, electrostatic interactions, and π-π stacking interactions [35,36,37]. Therefore, anion frameworks constructed via In(III) ions with linkers modified by functional groups conducive to efficiently and rapidly capture organic dyes from aqueous solution. However, the reported In-based MOFs constructed by [In(OOC)_4_]^−^ cluster and carboxylate linker often suffer from poor water stability, which seriously hinders their practical applications. On the other hand, ionic-functional MOFs with highly effective and selective adsorption of trace organic dye molecules are still not in the majority. Hence, it still remains a big challenge to rapid capture trace dye molecules from aqueous solution by using water-stable ionic-functional In-based MOFs.

Based on the above design guidance, we designed and synthesized a stable anionic porous MOF [Me_2_NH_2_]_2_In_2_[(TATAB)_4_(DMF)_4_]·(DMF)_4_(H_2_O)_4_ (HDU-1) from [In(OOC)_4_]^−^ cluster and a nitrogen-rich liker H_3_TATAB (4,4′,4″-*s*-triazine-1,3,5-triyltri-*p*-aminobenzoic acid) (Scheme S1), which can selectively capture and separate trace cationic organic dyes. The negatively charged [In(OOC)_4_]^−^ cluster and uncoordinated –COOH on the linker result in one unit cell of HDU-1 having 8 negative sites. The zeta potential of −20.8 mV dispersed in pure water also shows that HDU-1 possesses negatively charged surface potential. Therefore, the high electronegativity, water stability, and porosity of HDU-1 can facilitate the ion-exchange and Coulombic interaction. As expected, the HDU-1 exhibits high selectivity and removal rates towards trace cationic dyes with suitable size, such as methylene blue (MB), Brilliant green (BG), and Victoria blue B (VB) under the synergetic promotion of ion-exchange, Conulombic interaction, size-dependent, and π-π stacking interaction between host-guest.

## 2. Results and Discussion

**Figure 1:**
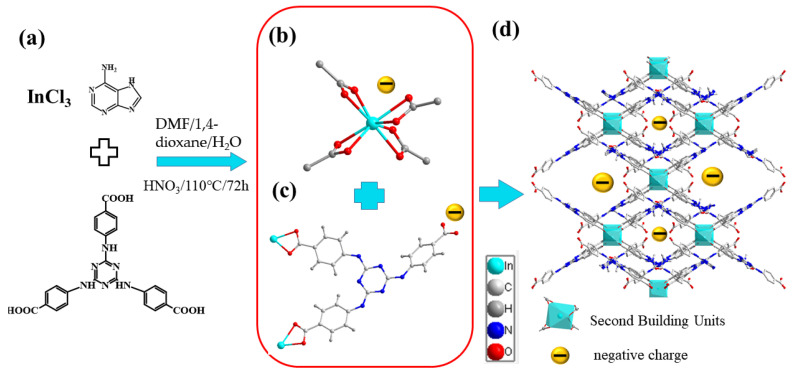
The synthesis process (**a**) and X-ray crystal structure of HDU-1: (**b**) the In(III) coordination mode; (**c**) the H_3_TATAB connected to two [In(COO)_4_] in non-planar; (**d**) the crystal structure viewed along the *c* axis.

**Figure 2:**
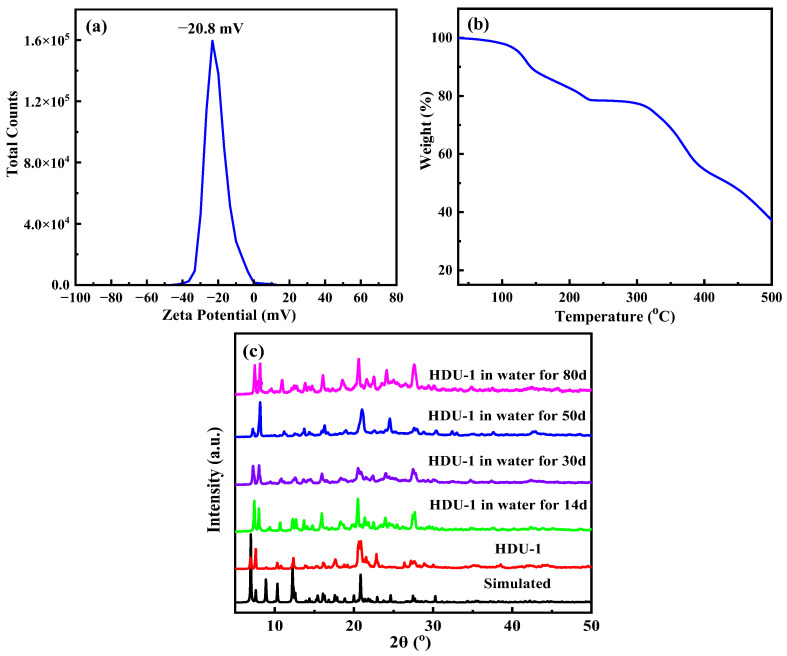
(**a**) The zeta potential of HDU-1 dispersed in pure water at room temperature, (**b**) thermogravimetric curves of HDU-1, (**c**) PXRD patterns of stability test of HDU-1 in water for different time.

Utilizing adenine as a guide agent, the HDU-1 was synthesized by solvent-thermal reaction choosing nitrogen-rich tricarboxylic acid ligand H_3_TATAB and InCl_3_ in DMF/H_2_O/1,4-dioxane for 3 days (Figure 1a). The scanning electron microscopy and optical photograph show that HDU-1 is a light yellow spindle crystal (Figure S1). The single-crystal X-ray diffraction (SCXRD) analyses revealed that HDU-1 crystallizes in the monoclinic space group *C*2/*c*, with a = 16.8151(12) Å, b = 19.9273(14) Å, c = 20.3388 Å, 𝛼 = γ = 90°, 𝛽 = 102°, V = 6642.5 Å^3^. Crystallographic data are summarized in Table 1. The asymmetric units of HDU-1 contain one crystallographically individual In^3+^ ion, two one-second TATAB^3−^ linkers, and one DMF molecule. Each In^3+^ ion is encircled by eight oxygen atoms from carboxyl groups of four deprotonated H_3_TATAB ligands to constitute [In(COO)_4_] secondary building units (SBUs) (Figure 1b) with In−O distances of 2.347, 2.252, 2.274, and 2.287 Å. Due to the flexibility of the -NH- between the benzene and triazine ring, the three benzene rings and triazine of the H_3_TATAB are not oriented in the same plane in the framework. The linker displays irregular shape and only two carboxyl groups for each ligand are coordinated by [In(COO)_4_] (Figure 1c). Thus, the symmetry of the whole framework reduces from tetragonal to monoclinic. Each [In(COO)_4_] cluster is connected by four linkers, and each linker is linked by two isolated [In(COO)_4_] clusters, to construct a 3D porous structure with uncoordinated carboxyl groups exposed to the channels (Figure 1d). There are a one-dimensional irregular channels along the *c* axis of approximately 3 × 6 Å^2^, taking into account the van der Waals radii. Noteworthily, the negatively charged behavior of the framework comes from a large amount of uncoordinated -COOH exposed to the channels and the negatively charged cluster [In(COO)_4_]^−^. The counteraction [Me_2_NH_2_]^+^ ions from decomposition of DMF solvent located within the channels are used to balance the negative charge of the skeleton. The zeta potential of −20.8 mV dispersed in pure water also shows that HDU-1 possesses negatively charged surface potential (Figure 2a). Moreover, the high density hydrogen bonds exist in channels which come from H atom on -NH- with O on DMF from channels, O on -COOH, and N on triazine ring, respectively (Figure S2).

The thermal stability for HDU-1 was proved by the thermogravimetric analysis with a heating rate 5 °C/min under N_2_ atmosphere. As shown in Figure 2b, the TGA curve shows that HDU-1 gradually loses solvent molecules of DMF and H_2_O before 225 °C, and is eventually thermally stable up to about 300 °C under N_2_ atmosphere. Finally, the metal oxides and amorphous carbon were formed. Elemental analysis for HDU-1 was measured and shows: Found (wt%): C, 51.27, N, 15.91, H, 5.06; Calcd (wt%): C, 51.05, N, 16.15, H, 4.83. The molecular formula of HDU-1 was determined as [Me_2_NH_2_]_2_In_2_ [(TATAB)_4_(DMF)_4_]·(DMF)_4_(H_2_O)_4_ by the chemical formula from crystal structure and elemental analysis (EA). The solvent content from molecular formula is consistent with the results of loss solvent molecules for TGA. As represented in Figure 2c, the PXRD peaks of as-synthesized HDU-1 are consistent with the simulated one obtained from single crystal data, indicating the pure phase of the obtained sample. HDU-1 was soaking in water for different time (14 d, 30 d, 50 d, and 80 d), aqueous solution with various pH value (2.3~12), and various phosphate buffered saline (PBS) solution (2~7.4) at room temperature to investigate the chemical stability (Figure 2c and Figure S3). It is found that the crystals could maintain the stability in various environments, which may be attributed to the large number of hydrogen bonds and the equilibrium ions in the framework.

**Figure 3:**
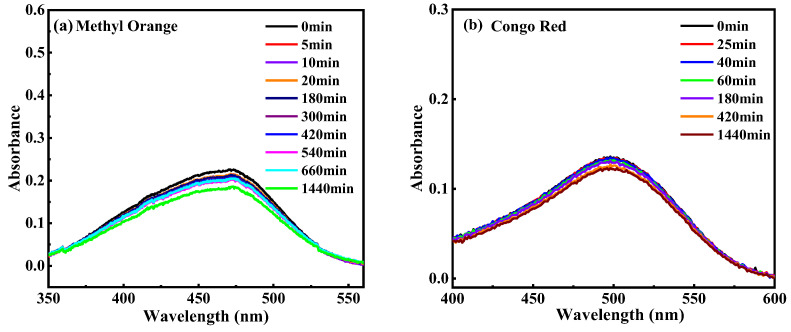
UV-Vis spectra of dyes (**a**) MO^−^ and (**b**) CR^−^ changed with time after adding HDU-1.

**Figure 4:**
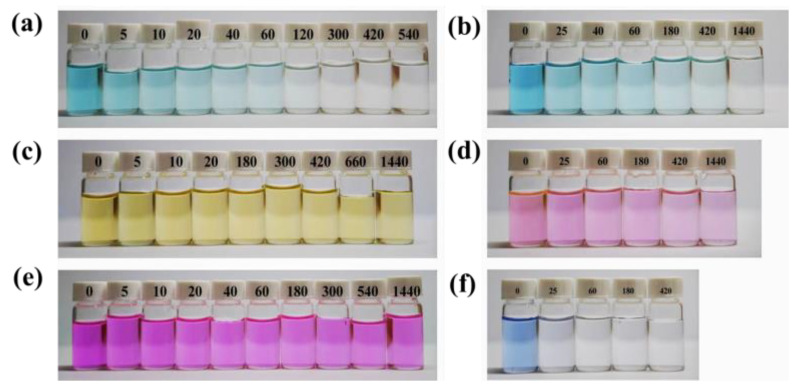
Photographs of the color change (**a**) BG^+^, (**b**) MB^+^, (**c**) MO^−^, (**d**) R6G^+^, (**e**) RhB^+^, and (**f**) VB^+^ aqueous solution during the dyes adsorption test with HDU-1 (unit: min).

**Figure 5:**
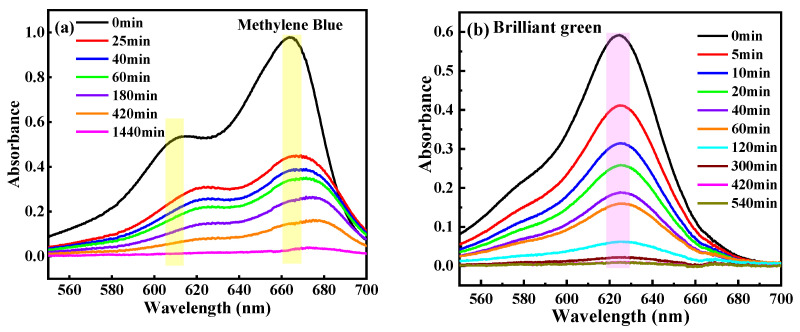

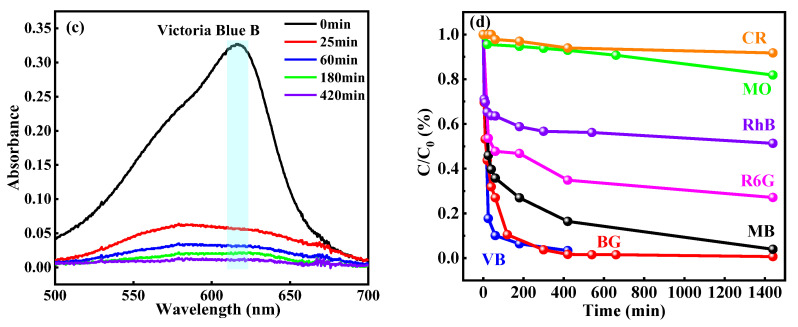
The UV-visible spectra for (**a**) MB^+^, (**b**) BG^+^, (**c**) VB^+^ during adsorption process with HDU-1 at times and (**d**) concentration changes of CR^−^, MO^−^, MB^+^, BG^+^, VB^+^, R6G^+^, and RhB^+^ in aqueous solution with time.

**Figure 6:**
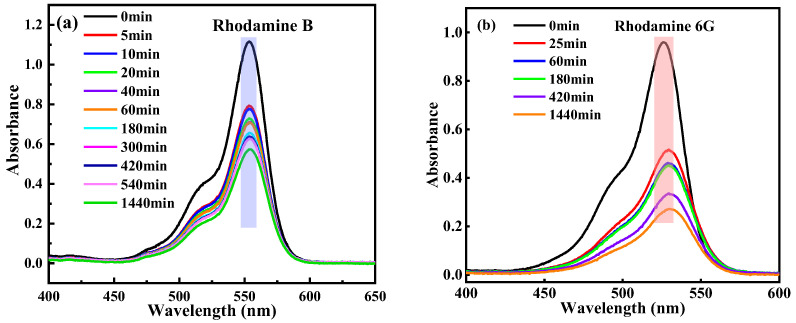
UV-Vis spectra of dyes (**a**) RhB^+^ and (**b**) R6G^+^ changed with time after adding HDU-1.

HDU-1 with the high-density electronegative sites and open/modified channels has a potential to remove and selectively separate the contaminant by ion-exchange and Coulombic interaction. We selected the different charged and configuration trace dyes (5 ppm) to evaluate the removal property of HDU-1, and the sizes and shapes of the dyes molecules as shown in Scheme S2. The methyl orange (MO^−^) and congo red (CR^−^) exhibits -1 charge and methylene blue (MB^+^), rhodamine B (RhB^+^), rhodamine 6G (R6G^+^), Brilliant green (BG^+^), and Victoria blue B (VB^+^) exhibits +1 charge, respectively. In addition, the sizes of five kinds of cationic dyes are totally different. A small amount of HDU-1 of 10 mg was immersed in a 15 mL various dyes aqueous solution. Then, the removal performance for organic dyes from aqueous solution was measured via UV-visible adsorption spectra. The results reveal that the anionic framework has no adsorption capacity at all for cationic dyes, such as MO^−^ and CR^−^ (Figure 3). There was no change in the color of the methyl orange solution (Figure 4). Among these five cationic organic dyes, HDU-1 can efficiently remove the MB^+^, BG^+^, and VB^+^ completely (Figure 5), and the removal processes can be observed by the color therewith disappeared of the solutions (Figure 4). While for the cationic dyes RhB^+^ and R6G^+^, obvious absorption peaks are still observed after 24 h, indicating that HDU-1 can only adsorb a fraction of RhB^+^ and R6G^+^ (Figure 4 and Figure 6).

In order to further evaluate the adsorption rates of HDU-1 for trace cationic organic dyes, the concentration changes of RhB^+^, R6G^+^ MB^+^, BG^+^, and VB^+^ in aqueous solution with time induced by HDU-1 are showed in Figure 4d. The removal rate of HDU-1 can reach 82.3% after 25 min, and the solution is nearly clarified after 180 min for VB^+^. While the MB^+^ and BG^+^ display just about 54.0% and 56.3%, removal efficiency after 25 min and 20 min, respectively. In addition, the removal efficiency of HDU-1 for MB and BG molecules is also comparable to those research in reported MOFs NOTT-210 [38], InOF [36], ZJU-24-0.89 [39], Cu_2_(L)(H_2_O)_2_ [40], and JOU-11 [20]. These results indicated that HDU-1 has different adsorption rate VB^+^ > BG^+^ > MB^+^ > RhB^+^ > R6G^+^ for the cationic organic dyes with different size and shape. The faster removal efficiency may be attributed to the stronger π-π interaction of the naphthalene ring on the Victoria blue B (VB^+^) with the linker of HDU-1. Although the peak intensity of PXRD patterns (Figure S4) of HDU-1 after dye-adsorption decreased, the structure of the crystals did not collapse during adsorption process. This is also proved by the fact that the adsorption peak of 1600 cm^−1^ for C=O stretching vibration of linker belonging to carboxyl group in the FT-IR spectroscopy does not change before and after dye-adsorption. The adsorption ability of HDU-1 for these trace cationic organic dyes may be attributed to the following aspects: (a) HDU-1 is anionic framework with high electronegative and surface potential can effective trap trace cationic organic dyes because of the Coulombic interaction and ion-exchange. (b) The open/modified channels with suitable size give opportunity to trap trace cationic organic dyes. (c) The π-π stacking interaction of aromatic rings between organic dye molecules and organic linker is conducive to adsorption. Furthermore, the pseudo-first order and pseudo-second order adsorption kinetics of MB^+^, BG^+^, VB^+^, R6G^+^, and RhB^+^ on HDU-1 are analyzed via the time-dependent adsorption capacity. As showed in Figure S6, the fitting results display that the kinetics data are better matched with the pseudo-second-order kinetic model.

**Figure 7:**
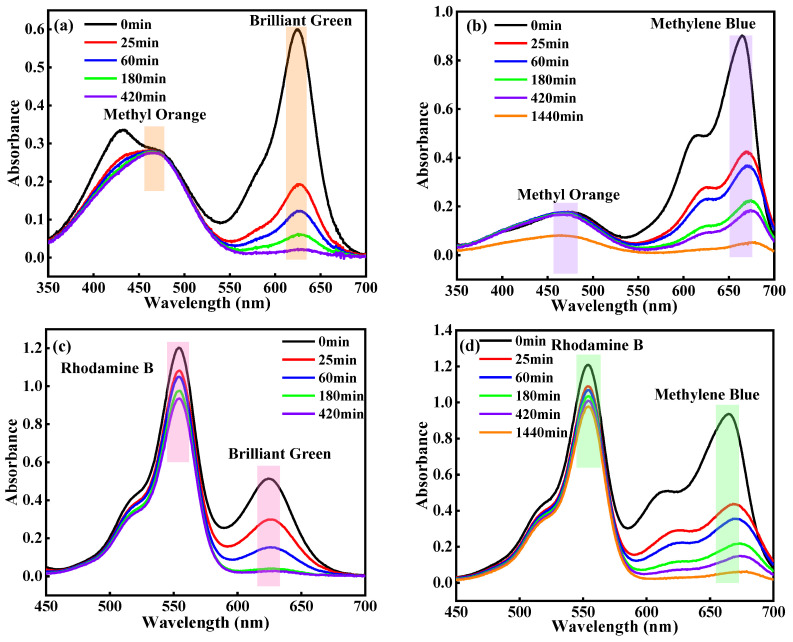
The UV-visible spectra for (**a**) MO^−^/BG^+^, (**b**) MO^−^/MB^+^, (**c**) BG^+^/RhB^+^, (**d**) MB^+^/RhB^+^ during adsorption process with HDU-1 at times.

**Figure 8:**
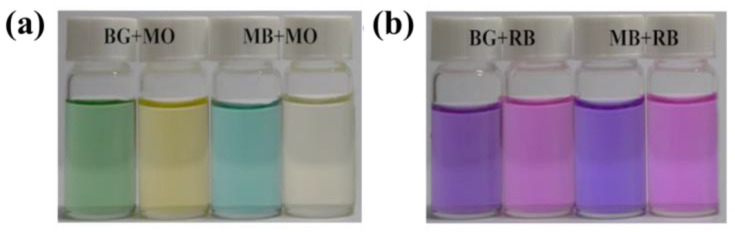
Photographs of the color change (**a**) MO^−^/BG^+^ and MO^−^/MB^+^, (**b**) BG^+^/RhB^+^, and MB^+^/RhB^+^ aqueous solution during the dyes adsorption test with HDU-1.

On the basis of the difference in dye adsorption capacity described above, the HDU-1 is capable of selectively separating dye molecules by two modes. The fresh prepared crystals were soaked in a mixed solution of two kind of dyes (1:1 in mole ratio). First, charge-dependent selective adsorption was investigated in a mixture of MO^−^/MB^+^ and MO^−^/BG^+^. As shown in Figure 7a, the trace cationic organic dyes were almost completely removed by HDU-1 (adsorption rate of 96.3% after 7 h for BG^+^ and 94.0% after 24 h for MB^+^), while the peak intensity of UV-visible spectra for MO^−^ after 7 h had no significant change. At the same time, the color of the mixed solution changed (Figure 8) also confirmed the selective adsorption of cationic organic dyes based on charge-dependent. The peak intensity of UV-visible spectra of MO^−^ still gone down after 24 h for the mixed MB^+^/MO^−^ solution, possibly due to the adsorption effect of adsorbed MB^+^ in channels on MO^−^ with small molecule size as time extends. Second, HDU-1 can selectivly remove the cationic organic dyes with different sizes and shape based on size-dependent mode. As shown in Figure 7, MB^+^ and BG^+^ were completely adsorbed while RhB^+^ were hardly removed because the molecular sizes of linear MB^+^ of 1.57 × 0.78 × 0.40 nm^3^ and subtriangular BG^+^ of 1.81 × 1.33 × 0.63 nm^3^ are smaller than that of RhB^+^ of 1.85 × 1.34 × 0.87 nm^3^. The color of the mixed MB^+^/RhB^+^ and BG^+^/RhB^+^ solution changed from purple to dark pink (Figure 8), demonstrating the selective adsorption of size-dependent. In a word, HDU-1 can be taken into account as a candidate adsorption porous material for the selective removal of cationic organic dye from wastewater.

The reusability plays an important role for the practical application of MOFs materials. In order to study the regeneration and reuse of HDU-1, we immersed the HDU-1 loading VB^+^ molecules in ethanol with a small amount of HCl (10 mM) for 24 h at room temperature to desorb VB^+^ dyes. As shown in Figure S7, it was observed the regeneration efficiency still achieves over 99% after three cycles. The regeneration performance of HDU-1 is further proved by the images of HDU-1 loading with dyes before and after elution (inset of Figure S7a), which confirms that the HDU-1 can be an ideal renewable porous material.

## 3. Experimental

### 3.1. Materials and Methods

All chemicals were obtained from commercial sources and used without additional purification. H_3_TATAB (4,4′,4″-*s*-triazine-1,3,5-triyltri-*p*-aminobenzoic acid, 98%) was purchased from Beijing Hwrkchemical Co,. Ltd., Beijing, China. *N*,*N*-Dimethylformamide (C_3_H_7_NO, AR, 99.5%), Dioxane (C_4_H_8_O_2_, for HPLC, ≥99.5%), Methylene Blue (C_16_H_18_ClN_3_S, ≥70%), Adenine (C_5_H_5_N_5_, 98%), Victoria blue B (C_33_H_32_ClN_3_, 80%), Brilliant Green (C_27_H_34_N_2_O_4_S, 95%), and InCl3 (99.99%) were purchased from Shanghai Aladdin Biochemical Technology Co., Ltd., Shanghai, China. Rhodamine B (C_28_H_31_ClN_2_O_3_, AR) and Rhodamine 6G (C_28_H_31_N_2_O_3_Cl, AR) were purchased from Shanghai Rhawn Chemical Technology Co., Ltd., Shanghai, China. Nitric acid (HNO_3_, 65–68%) was purchased from Chinasun Specialty Products Co., Ltd., Suzhou, China. Cango Red (C_32_H_22_N_6_Na_2_O_6_S_2_, >98.0%) was purchased from Sinopharm Chemical Reagent Co., Ltd., Shanghai, China. Methyl Orange (C_14_H_14_N_3_NaO_3_S, 96%) was purchased from Shanghai Richjoint Chemical Reagent Co., Ltd., Shanghai, China.

Thermogravimetric analyses (TGA) were carried out on a Netzsch TGA 209 F3 with a heating rate of 5 °C/min in N_2_ atmosphere. Elemental analyses for C, H, and N were carried out on a Flash EA1112 micro elemental analyzer. Powder X-ray diffraction (PXRD) patterns were recorded in the 2*θ* range from 5 to 50° on a Shimadzu XRD 7000 diffractometer with Cu K*_α_*(λ = 1.542 Å) radiation at room temperature. Infrared spectrum (IR) was collected in the wavenumber range from 4000 to 500 cm^−1^ on Thermo Fisher Nicolet iS10 spectrometer using KBr pallets. The zeta potential was carried out on Malvern Zetasizer Nano ZS90. The UV−vis spectra were performed in the wavelength range from 250 to 800 nm on a Shimadzu UV-3600 spectrophotometer.

### 3.2. Syntheses of HDU-1

H_3_TATAB (0.1945 g), InCl_3_ (0.0133 g), and adenine (0.0122 g) were dissolved in a glass vial containing 6.2 mL of the mixed solution *N*,*N*′-dimethylformamide (DMF)/1,4-dioxane/H_2_O (4:1:1.2), and adding 4 drops of HNO_3_ solution of 1 M, then the mixed solution was placed into the Teflon-lined stainless steel autoclave and heated at 110 °C for 3 days. The light yellow crystals were gathered by filtering and washed three times with DMF and dried in air at room temperature.

### 3.3. X-ray-Crystallography

The single crystal measurement of HDU-1 was executed on Bruker APEX-II diffractometer coupled to CCD detector with graphite-monochromatic Mo Kα radiation (λ = 0.71073 Å) and Atlas detector at 273 K. The unit cell parameters and data were determined and collected directly by CrysAlisPro program. The structure of HDU-1 was solved by the direct methods and refined by the full matrix least square method of SHELX program package. All non-hydrogen atoms were directly located according to Fourier diffraction points and refined by anisotropy. H atoms on the ligand were added by theoretical model. The disordered solvent molecules and ions in the crystal channel were computed by PLATON software/SQUEEZE subroutine CCDC: 2159854.

### 3.4. Dye Adsorption Measurements

Seven different organic dyes with different electric charge, kinetic diameter, and molecular structure were selected to explore the adsorption property and adsorption mechanism of HDU-1. In order to study adsorption kinetic, HDU-1 was dried overnight under vacuum at 25 °C and kept in a desiccator. Then, the fresh prepared crystals of 10 mg were weighed precisely. Then, the adsorbents were added into the solution (15 mL) of methyl orange (MO^−^), congo red (CR^−^) methylene blue (MB^+^), rhodamine B (RhB^+^), rhodamine 6G (R6G^+^), Brilliant green (BG^+^), and Victoria blue B (VB^+^) with the concentration of 5 ppm. The mixture was placed in centrifuge tube and maintained in shaker for a fixed time (5 min to 24 h) at 30 °C. The syringe filter (PTFE, hydrophobic, 0.22 μm) was used to separate the solution from the adsorbent. The UV-vis curves of the remaining dye solution were performed on Shimadzu UV-3600 spectrophotometer. For the dyes separation test, two different dyes of 15 mL with the concentration of 5 ppm were mixed and the adsorbent of 10 mg was added into the mixed solution. The chemical structures of dyes are shown in Scheme S2.

## 4. Conclusions

In summary, an anionic functional metal-organic frameworks HDU-1 has been designed and constructed from a nitrogen-rich tricarboxylic acid ligand and In(III) ion. The HDU-1 possesses high-density electronegative sites and uncoordinated–COOH on the linker. The zeta potential of −20.8 mV dispersed in pure water also shows that HDU-1 possesses negatively charged surface potential. The negative charge nature and porosity of HDU-1 can facilitate the ion-exchange and Coulombic interaction. Our results exhibit that HDU-1 can effectively remove the trace cationic organic dyes of MB^+^, VB^+^, and BG^+^ from aqueous solution based on charge-dependent, size-dependent, and π-π stacking interaction between host-guest. The removal rate of HDU-1 can reach 82.3% after 25 min, and the solution is nearly clarified after 180 min for VB^+^. At the same time, MB^+^ and BG^+^ can be separated by HDU-1 from the mixed solution of MO^−^/BG^+^, MO^−^/MB^+^, BG^+^/RhB^+^, MB^+^/RhB^+^. Hence, HDU-1 can be taken into account as a candidate adsorption porous material for the selective removal of cationic organic dye from wastewater.

## Figures and Tables

**Table 1:** Crystallographic Data Collection and Refinement Results for HDU-1.

	HDU-1
Chemical formula	C_108_H_92_In_2_O_28_N_28_
Formula weight	2459.73
Temperature (K)	273(2)
Wavelength (Å)	0.71073
Crystal system	Monoclinic
Space group	*C* 2*/c*
*a* (Å)	16.8151(12)
*b* (Å)	19.9273(14)
*c* (Å)	20.3388(14)
*V* (Å^3^)	6642.5(8)
α	90
β	102
γ	90
*Z*	4
Density (calculated g/cm^3^)	1.230
Absorbance coefficient (mm^−1^)	0.422
*F*(000)	2516
Crystal size (mm^3^)	0.3 × 0.21 × 0.12
Goodness of fit on *F*^2^	1.099
*R*_1_, *wR*_2_ [*I* > 2*σ*(*I*)]	0.0762, 0.1802
*R*_1_, *wR*_2_ (all data)	0.0790, 0.1814
Largest difference peak and hole(e/Å^3^)	1.097, −2.408

K is Kelvin for the unit of temperature, Z is the number of molecules contained in each cell, *R*_1_ = Σ(|*F_o_*| − |*F_c_*|)/Σ|*F_o_*|; *wR*_2_ = |Σ*w*(|*F_o_*| − |*F_c_*|^2^)/Σ*wF_o_*^2^]^1/2^.

## Data Availability

The data presented in this study are available on request from the corresponding authors.

## Supplementary Materials

The following supporting information can be downloaded at: https://www.mdpi.com/article/10.3390/molecules28134980/s1, Scheme S1: Chemical structure of H_3_TATAB; Scheme S2. Molecular structures of seven organic dyes; Figure S1: (a) SEM image of HDU-1 and (b) optical photograph of HDU-1; Figure S2: The hydrogen bonds which come from H atom on -NH- with O on DMF from channels (a), O on -COOH (b) and N on triazine ring (c), respectively; Figure S3. PXRD patterns of stability test of HDU-1 in pH solution for different time and PBS solution for 24; Figure S4. PXRD comparison of HDU-1 before and after adsorption of dyes; Figure S5. FT-IR comparison of HDU-1 before and after adsorption of dyes; Figure S6. The pseudo-first order and pseudo-second order adsorption kinetics of MB^+^, BG^+^, VB^+^, R6G^+^ and RhB^+^ on HDU-1 via the time-dependent adsorption capacity; Figure S7. (a) The UV-vis spectra of VB absorption/desorption of HDU-1 (inset: image of VB solution after adsorption/desorption for four times), (b) Regeneration performance of HDU-1 for VB adsorption; Table S1. Molecular size of the dyes.
