# Supplementary material for: An Anionic Porous Indium-Organic Framework with Nitrogen-Rich Linker for Efficient and Selective Removal of Trace Cationic Dyes
Source: Molecules. 2023 Jun 25;28(13):4980. doi: 10.3390/molecules28134980 (PMC10343176; doi:10.3390/molecules28134980)
Supplement: Supplementary file 1 [file molecules-28-04980-s001.zip › molecules-2403813-supplementary.docx]

*Supplementary Information***An anionic porous indium-organic framework with nitrogen-rich linker for efficient and selective removal of trace cationic dyes**

**Lihui Feng ^1^, Xiaofei Zhang ^1^, Zhekuang Jin ^1^, Jiashang Chen ^1^, Xing Duan ^1,^*, Shiyu Ma ^1^, Zhe Kong ^1,^*and Tifeng Xia ^2,^***

^1^ Center of Advanced Optoelectronic Materials and Devices, Key Laboratory of Novel Materials for Sensor of Zhejiang Province, College of materials & environmental engineering, Hangzhou Dianzi University, Hangzhou 310018, China.; star1987@hdu.edu.cn; zhekong@hdu.edu.cn

^2^ Institute of Materials, China Academy of Engineering Physics, Mianyang 621907, China.; xiatifeng@caep.cn

***** Correspondence: [star1987@hdu.edu.cn](mailto:star1987@hdu.edu.cn); [zhekong@hdu.edu.cn](mailto:zhekong@hdu.edu.cn); xiatifeng@caep.cn

**Scheme S1.** Chemical structure of H_3_TATAB.

**Scheme S2.** Molecular structures of seven organic dyes.

**
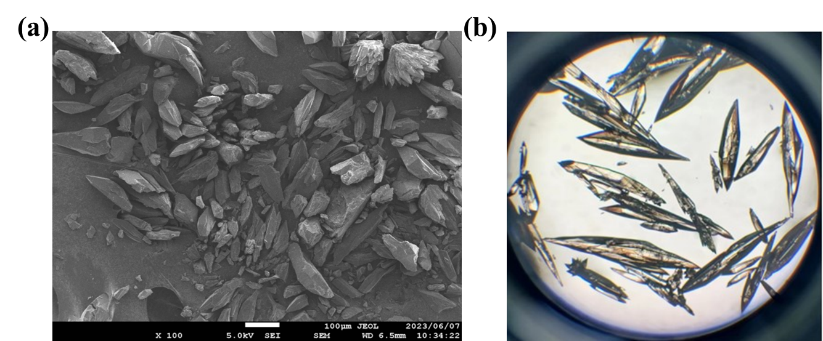
**

**Figure S1** (a) SEM image of HDU-1 and (b) optical photograph of HDU-1


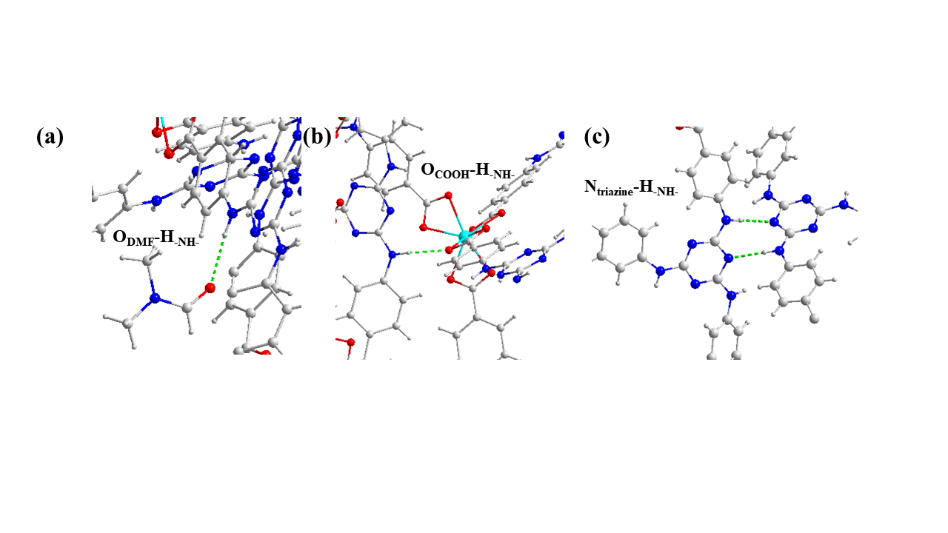


**Figure S2.** The hydrogen bonds which come from H atom on -NH- with O on DMF from channels (a), O on -COOH (b) and N on triazine ring (c), respectively.

**Figure S3.** PXRD patterns of stability test of HDU-1 in pH solution for different time and PBS solution for 24.

**Figure S4.** PXRD comparison of HDU-1 before and after adsorption of dyes.

**Figure S5.** FT-IR comparison of HDU-1 before and after adsorption of dyes.

**Figure S6** The pseudo-first order and pseudo-second order adsorption kinetics of MB^+^, BG^+^, VB^+^, R6G^+^ and RhB^+^ on HDU-1 via the time-dependent adsorption capacity.

**Figure S7** (a) The UV-vis spectra of VB absorption/desorption of HDU-1 (inset: image of VB solution after adsorption/desorption for four times), (b) Regeneration performance of HDU-1 for VB adsorption.

**Table S1.** Molecular size of the dyes

| Dyes | chemical formula | molecular size (nm^3^) |
| --- | --- | --- |
| Methylene Blue | C_16_H_18_ClN_3_S | 1.5678 × 0.7796 × 0.4022 |
| Victoria blue B | C_33_H_32_ClN_3_ | 1.7582 × 1.454 × 0.6587 |
| Brilliant Green | C_27_H_34_N_2_O_4_S | 1.8104 × 1.3288 × 0.629 |
| Rhodamine B | C_28_H_31_ClN_2_O_3_ | 1.8523 × 1.3409 × 0.8655 |
| Rhodamine 6G | C_28_H_31_N_2_O_3_Cl | 1.5411 × 1.4158 × 0.922 |
| Cango Red | C_32_H_22_N_6_Na_2_O_6_S_2_ | 2.8682 × 1.0653 × 0.9169 |
| Methyl Orange | C_14_H_14_N_3_NaO_3_S | 1.9543 × 0.7179 × 0.5971 |
